# Chikungunya Virus Strains from Each Genetic Clade Bind Sulfated Glycosaminoglycans as Attachment Factors

**DOI:** 10.1128/JVI.01500-20

**Published:** 2020-11-23

**Authors:** Nicole McAllister, Yan Liu, Lisete M. Silva, Anthony J. Lentscher, Wengang Chai, Nian Wu, Kira A. Griswold, Krishnan Raghunathan, Lo Vang, Jeff Alexander, Kelly L. Warfield, Michael S. Diamond, Ten Feizi, Laurie A. Silva, Terence S. Dermody

**Affiliations:** a Department of Microbiology and Molecular Genetics, University of Pittsburgh School of Medicine, Pittsburgh, Pennsylvania, USA; b Center for Microbial Pathogenesis, UPMC Children’s Hospital of Pittsburgh, Pittsburgh, Pennsylvania, USA; c Glycosciences Laboratory, Department of Metabolism, Digestion and Reproduction, Imperial College London, London, United Kingdom; d LAQV-REQUIMTE, Department of Chemistry, University of Aveiro, Aveiro, Portugal; e Division of Histology and Embryology, Medical College, Jinan University, Guangzhou, China; f Department of Pediatrics, University of Pittsburgh School of Medicine, Pittsburgh, Pennsylvania, USA; g Emergent Travel Health, Emergent BioSolutions Inc., San Diego, California, USA; h Department of Pathology and Immunology, Washington University School of Medicine, St. Louis, Missouri, USA; i Department of Medicine, Washington University School of Medicine, St. Louis, Missouri, USA; j Department of Molecular Microbiology, Washington University School of Medicine, St. Louis, Missouri, USA; Loyola University Chicago

**Keywords:** attachment factors, glycan microarrays, glycosaminoglycans, heparan sulfate, alphavirus, chikungunya virus, glycans

## Abstract

Chikungunya virus (CHIKV) is an arthritogenic alphavirus that causes debilitating musculoskeletal disease. CHIKV displays broad cell, tissue, and species tropism, which may correlate with the attachment factors and entry receptors used by the virus. Cell surface glycosaminoglycans (GAGs) have been identified as CHIKV attachment factors. However, the specific types of GAGs and potentially other glycans to which CHIKV binds and whether there are strain-specific differences in GAG binding are not fully understood. To identify the types of glycans bound by CHIKV, we conducted glycan microarray analyses and discovered that CHIKV preferentially binds GAGs. Microarray results also indicate that sulfate groups on GAGs are essential for CHIKV binding and that CHIKV binds most strongly to longer GAG chains of heparin and heparan sulfate. To determine whether GAG binding capacity varies among CHIKV strains, a representative strain from each genetic clade was tested. While all strains directly bound to heparin and chondroitin sulfate in enzyme-linked immunosorbent assays (ELISAs) and depended on heparan sulfate for efficient cell binding and infection, we observed some variation by strain. Enzymatic removal of cell surface GAGs and genetic ablation that diminishes GAG expression reduced CHIKV binding and infectivity of all strains. Collectively, these data demonstrate that GAGs are the preferred glycan bound by CHIKV, enhance our understanding of the specific GAG moieties required for CHIKV binding, define strain differences in GAG engagement, and provide further evidence for a critical function of GAGs in CHIKV cell attachment and infection.

**IMPORTANCE** Alphavirus infections are a global health threat, contributing to outbreaks of disease in many parts of the world. Recent epidemics caused by CHIKV, an arthritogenic alphavirus, resulted in more than 8.5 million cases as the virus has spread into new geographic regions, including the Western Hemisphere. CHIKV causes disease in the majority of people infected, leading to severe and debilitating arthritis. Despite the severity of CHIKV disease, there are no licensed therapeutics. Since attachment factors and receptors are determinants of viral tropism and pathogenesis, understanding these virus-host interactions can enhance our knowledge of CHIKV infection. We analyzed over 670 glycans and identified GAGs as the main glycan bound by CHIKV. We defined specific GAG components required for CHIKV binding and assessed strain-specific differences in GAG binding capacity. These studies provide insight about cell surface molecules that CHIKV binds, which could facilitate the development of antiviral therapeutics targeting the CHIKV attachment step.

## Abstract (precis)

Alphavirus infections are a global health threat, contributing to outbreaks of disease in many parts of the world. Recent epidemics caused by CHIKV, an arthritogenic alphavirus, resulted in more than 8.5 million cases as the virus has spread into new geographic regions, including the Western Hemisphere. CHIKV causes disease in the majority of people infected, leading to severe and debilitating arthritis. Despite the severity of CHIKV disease, there are no licensed therapeutics. Since attachment factors and receptors are determinants of viral tropism and pathogenesis, understanding these virus-host interactions can enhance our knowledge of CHIKV infection. We analyzed over 670 glycans and identified GAGs as the main glycan bound by CHIKV. We defined specific GAG components required for CHIKV binding and assessed strain-specific differences in GAG binding capacity. These studies provide insight about cell surface molecules that CHIKV binds, which could facilitate the development of antiviral therapeutics targeting the CHIKV attachment step.

## INTRODUCTION

To initiate infection, viruses interact with a variety of cell surface molecules, including proteins, carbohydrates, and lipids (1, 2). Binding to abundantly expressed cell surface molecules, which are sometimes called attachment factors, concentrates viral particles at the plasma membrane, which enhances the probability of engagement with an entry receptor (2). The interaction between a virus and an attachment factor is usually of low affinity (2). In contrast, interactions with entry receptors are usually of high affinity and often trigger conformational changes in viral surface proteins that promote viral entry (2). Expression of attachment factors and entry receptors is often a determinant of viral tropism and can influence disease (3), making it important to identify these host factors and characterize their function in viral replication. When multiple attachment factors or entry receptors are used by a virus, defining the function of each during viral infection can be complex. Overall, the molecular mechanisms by which viruses bind to host cells and how such virus-receptor interactions influence tropism and disease are still not completely understood, especially for emerging viruses.

Mosquito-transmitted alphaviruses are a global health threat and periodically reemerge to cause epidemics of disease in many parts of the world (4). Alphavirus introductions into naive populations have resulted in large epidemics, such as the chikungunya virus (CHIKV) epidemics that began in 2004 and 2013, which collectively resulted in more than 8.5 million cases and the spread of the virus into new geographic regions, including the Western Hemisphere (5–11). These epidemics were caused by CHIKV strains from two of the three genetically distinct CHIKV clades (the East Central South African [ECSA] and Asian clades, respectively) (12, 13), while strains from the third clade (West African) have remained endemic to western Africa (6). CHIKV causes disease in approximately 80% of those infected (14, 15), with manifestations commonly including fever, rash, myalgia, arthralgia, and arthritis (16, 17). CHIKV disease is usually self-limited and rarely fatal, but infection can cause acute and chronic disabilities that impair quality of life (18). Up to 60% of infected individuals experience debilitating arthralgia and arthritis that persist for months to years after infection (16, 17). Additionally, large CHIKV epidemics have severe social and economic consequences (19). Despite the severity of CHIKV disease, there are no licensed antivirals or vaccines.

CHIKV can infect mosquitoes, nonhuman primates, and humans (20). In mosquitoes, CHIKV replicates in the midgut, salivary glands, fat bodies, and ovaries (21, 22). While CHIKV replicates in many human cell lines, including fibroblasts (23), macrophages (24), keratinocytes (25), epithelial cells (23), muscle cells (23, 26), and endothelial cells (23), the cells and tissues targeted in infected humans are less well defined. However, studies using mice demonstrate CHIKV dissemination into a variety of tissues, including dermis, lymph nodes, spleen, muscle, joints, and tendons (27–30). The broad cell, tissue, and species tropism observed for CHIKV may correlate with the expression of attachment factors or entry receptors used by the virus.

Several cell surface molecules have been identified to facilitate CHIKV attachment and entry. CHIKV binds Mxra8 as an entry receptor (31, 32), but absent or decreased expression of Mxra8 in several cell types does not completely abrogate CHIKV infection, suggesting that CHIKV can use other entry receptors (31). Additionally, a variety of cell surface molecules may act as attachment factors for CHIKV (33–37), including glycosaminoglycans (GAGs) (38–41). GAGs serve as attachment factors for many pathogenic viruses (38–40, 42–53) and are expressed ubiquitously in humans and mosquitoes (54–56). GAGs are negatively charged linear polysaccharides composed of repeating disaccharide units expressed at the cell surface and in the extracellular matrix (54). Interactions with GAGs are often mediated by positively charged amino acid side chains of protein ligands (57). There are four main types of GAGs based on differences in their repeating disaccharide units, including heparin/heparan sulfate (HS), chondroitin sulfate (CS)/dermatan sulfate (DS), keratan sulfate (KS), and hyaluronan (54). With the exception of hyaluronan, the other types of GAGs are highly sulfated (54). Variations in GAG chain length and degree and pattern of sulfation are determined by the expression and relative abundance of specific GAG biosynthetic enzymes (54, 58). Although heparin and HS are structurally similar, heparin is a more highly sulfated version of HS, composed of more iduronic acid, and is often used experimentally instead of HS due to accessibility and cost (54, 59). HS and CS/DS are abundantly expressed at the sites CHIKV infects. In mosquitoes, HS and CS/DS are expressed in the ovaries, midgut, and salivary glands (56, 60, 61). In mammals, HS is expressed primarily on epithelial cells, fibroblasts, endothelial cells, skin, and muscle (54, 62–64), and CS/DS is found mainly in cartilage, connective tissue, fibroblasts, macrophages, and endothelial cells (54, 65). Thus, HS and CS/DS expression overlaps with the broad cell and tissue tropism of the virus.

Cell culture adaptation of CHIKV, which often results in mutations in the E2 attachment protein, can enhance GAG binding (66). CHIKV strain 181/25 displays increased GAG binding due to a specific mutation in E2 (G82R) (38, 39) that was acquired after 29 passages in cell culture (67, 68). However, for at least some field isolate strains, efficient infection in cell culture depends on GAG expression (38–41). Accordingly, preincubation of some CHIKV strains with soluble GAGs prior to cell adsorption inhibits infection *in vitro* (38, 40). It is not clear whether CHIKV preferentially binds to different GAG types or whether CHIKV strains from the three genetically distinct clades differ in GAG binding. Moreover, the requirement of specific GAGs for CHIKV binding and infection of cells with various levels of GAG and Mxra8 expression has not been defined.

In this study, we used microarrays to identify glycans bound by CHIKV. We discovered that CHIKV preferentially binds GAGs relative to other glycan types tested and identified heparin and HS to be bound by CHIKV most efficiently. We found that human- and mosquito-isolated CHIKV strains from each CHIKV clade directly bind to GAGs and require HS for efficient binding and infection. Although CHIKV directly binds to CS chains, CS is not required for infection and influences binding for only some strains in the cells tested. The requirement of sulfated GAGs for CHIKV binding and infection was inversely correlated with the levels of Mxra8 expression. Finally, strains of each CHIKV clade displayed differences in the efficiency of GAG utilization. These studies suggest that HS and, to a lesser extent, possibly CS/DS function as a CHIKV attachment factor in the presence and absence of the Mxra8 entry receptor. Collectively, these data enhance our understanding of attachment factor engagement for diverse CHIKV strains.

## RESULTS

### CHIKV directly and preferentially binds sulfated GAGs.

**FIG 1:**
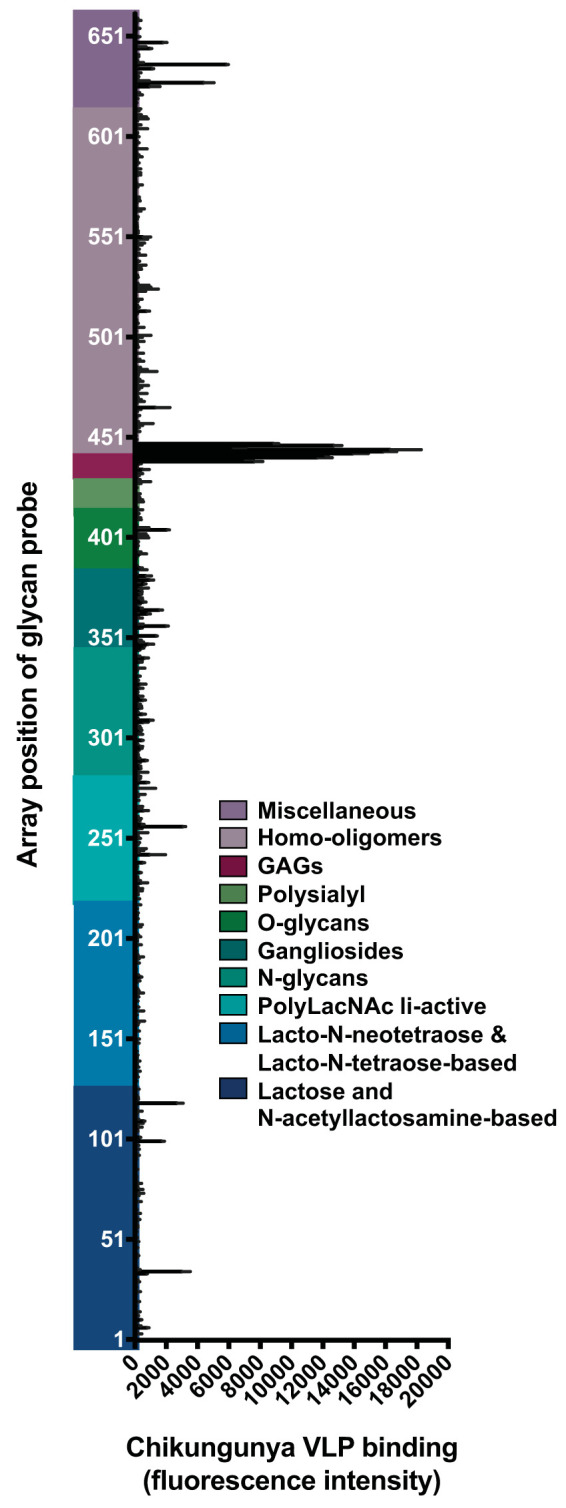
Chikungunya VLPs bind specifically to GAGs. A glycan microarray composed of 672 lipid-linked glycan probes was incubated with purified chikungunya virus-like particles (VLPs) (50 μg/ml). Bound VLPs were fixed with 4% PFA and detected using an anti-CHIKV E2-specific monoclonal antibody (CHK-152), followed by biotin-conjugated IgG and streptavidin-conjugated Alexa Fluor 647. VLP-glycan binding is reported as the mean fluorescence intensity of duplicate spots of each lipid-linked glycan probe printed at 5 fmol. The glycan groups tested are arranged according to their backbone sequences as annotated. The glycans tested, probe sequences, and binding intensities are listed in Table S1 in the supplemental material. Binding data shown are representative of two independent experiments. Error bars represent half of the difference between the two values.

Some strains of CHIKV bind directly to heparin *in vitro* (38, 39). To identify other glycans to which CHIKV binds, we conducted glycan microarray analyses using virus-like particles (VLPs). Chikungunya VLPs are structurally indistinguishable from native chikungunya virions (69) and can be used in experiments at a lower biosafety level than for pathogenic CHIKV. The VLPs used in our experiments are composed of the structural proteins of West African clade CHIKV strain 37997 (70) and are currently in advanced development as a vaccine candidate by Emergent BioSolutions (71–73). The microarray contained 672 sequence-defined lipid-linked oligosaccharides, representing the major types of mammalian glycans found on glycoproteins, glycolipids, and proteoglycans, as well as those derived from glucan polysaccharides of bacteria, fungi, and plants (Fig. 1; see Table S1 in the supplemental material). Ten heparin-derived oligosaccharides (2-mer to 20-mer chains) were included in this array as representatives of GAG-related sequences (Table S1). Chikungunya VLPs were overlaid onto the microarray, and VLP binding was detected by indirect immunofluorescence.

Among the 672 glycans tested in the microarray, approximately 30 glycans showed a VLP binding signal above background (Fig. 1 and Table S1). The 10 highest VLP binding signals were produced by heparin GAGs of various lengths (Fig. 1 and Table S1), suggesting that GAGs are the preferred glycan type bound by CHIKV. Binding was observed with a heparin 2-mer, and binding signals increased with increasing length of heparin chains (Table S1). Among the non-GAGs bound, most are negatively charged, including a “ring-opened” NeuAc monosaccharide (position 637), SU-3GlcAβ-3Galβ-4Glc (position 36), and Carra-Hexa-4S (position 669) (Fig. 1 and Table S1). Collectively, these data demonstrate that GAGs are preferentially bound by chikungunya VLPs *in vitro* and highlight a potential role for GAG chain length in the efficiency of virus binding.

**FIG 2:**
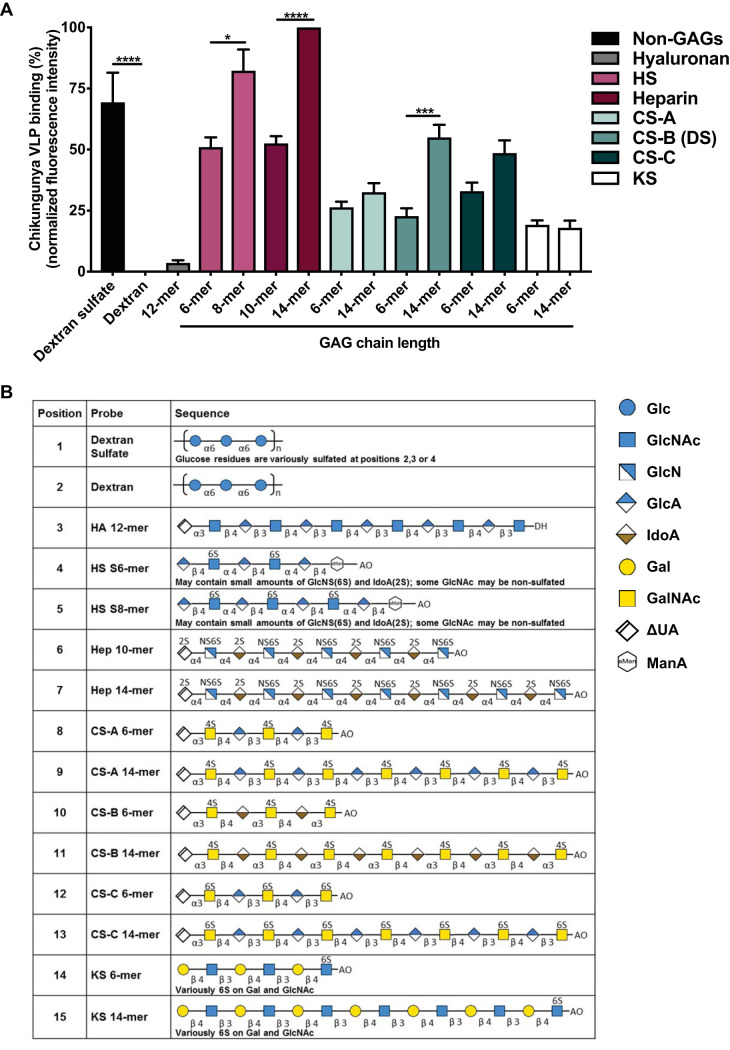
Chikungunya VLPs bind to longer, sulfated iduronic acid-containing GAGs with a preference for heparin and HS. A GAG-focused microarray composed of GAGs differing in length (indicated by a number with -mer) and sulfation was incubated with chikungunya VLPs. Dextran and dextran sulfate, non-GAG glycans, also were included in the array to assess sulfation requirements for binding. (A) Chikungunya VLPs were incubated on the microarray. Bound VLPs were fixed with 4% PFA and detected using either an anti-CHIKV E2-specific monoclonal antibody (CHK-152) or anti-CHIKV ascites fluid, followed by biotin-conjugated IgG and streptavidin-conjugated Alexa Fluor 647. VLP-glycan binding is normalized to heparin 14-mer fluorescence intensity signals. Fluorescence intensity was determined from duplicate spots of each glycan probe printed at 5 fmol for GAG NGL probes and 0.1 ng for dextran and dextran sulfate. Binding data shown are an average from five independent experiments, except for results with HS, which are from three independent experiments. Error bars indicate standard error of the mean (SEM). *P* values were determined by one-way analysis of variance (ANOVA) followed by Tukey’s multiple-comparison test (*, *P < *0.05; ***, *P < *0.001; ****, *P < *0.0001). Statistics presented within the graph indicate statistical significance only between samples of each glycan type. (B) The backbone sequences for each glycan probe used on the microarray are listed. Glc, glucose; GlcNAc, *N*-acetylglucosamine; GlcN, glucosamine; GlcA, glucuronic acid; IdoA, iduronic acid; Gal, galactose; GalNAc, *N*-acetylgalactosamine; ΔUA, 4,5-unsaturated hexuronic acid; ManA, 2,5-anhydro-mannose; DH and AO, lipid moieties of NGLs prepared by reductive amination and oxime ligation, respectively. Further details are in Tables S2 and S3 in the supplemental material.

To gain additional information about the GAG binding specificities of CHIKV, we used GAG-focused microarrays. These microarrays included 15 size-defined oligosaccharides derived from different types of GAGs: heparin, HS, CS-A, CS-B (DS), CS-C, KS, and hyaluronan, which was the only nonsulfated GAG in this analysis (Fig. 2; see Table S2 in the supplemental material). Short (6- or 10-mer) and long (up to 14-mer) chains were included for each GAG type except the hyaluronan 12-mer, HS 6-mer, and HS 8-mer (Fig. 2 and Table S2). Larger size-defined fractions of HS oligosaccharides were not available for the study due to the sequence heterogeneity of HS relative to other GAG types. Two non-GAG polysaccharides, dextran sulfate and dextran (74), also were included as controls for highly sulfated and neutral saccharides, respectively. Chikungunya VLPs were overlaid onto the GAG-focused array, and VLP binding was detected by indirect immunofluorescence.

Whereas VLPs bound to dextran sulfate, binding to unsulfated dextran was not detected, and very little binding to hyaluronan, an unsulfated GAG, was observed (Fig. 2A). These data suggest an important function for sulfation in CHIKV-glycan interactions. VLPs bound all sulfated GAGs above background with various intensities (Fig. 2A). The strongest binding signals were observed with heparin, followed by CS-B, CS-C, CS-A, and weakest for KS (Fig. 2A). In general, stronger binding signals were observed with longer GAG oligosaccharides, especially with the heparin 14-mer, HS 8-mer, and CS-B 14-mer, which all reached statistical significance. Interestingly, the GAGs bound most strongly by CHIKV, including heparin, HS, and CS-B (DS), all contain iduronic acid, while the other GAG types do not (54) (Fig. 2B), suggesting that iduronic acid contributes to CHIKV binding. Overall, CHIKV binds with greatest avidity *in vitro* to longer, sulfated chains of GAGs, with a preference for HS and heparin.

### Multiple CHIKV strains directly bind heparin and CS.

To determine whether GAG binding efficiency differs between CHIKV strains and to validate the microarray results, we assessed viral binding to heparin and CS by enzyme-linked immunosorbent assay (ELISA). Three CHIKV strains, SL15649 (29), H20235 (75), and 37997 (70), were selected to represent the three CHIKV genetic clades (ECSA, Asian, and West African, respectively) (Table 1). Importantly, the strains chosen for analysis were isolated from infected humans or mosquitoes and were minimally passaged in cell culture prior to sequencing and construction of infectious cDNA clones (Table 1). We used CHIKV strain 181/25 as a positive control for heparin binding. Strain 181/25 was derived from plaque-to-plaque passaging of parental strain AF15561 of the Asian CHIKV clade (67, 68). Cell culture adaptation of 181/25 led to mutations in the E2 attachment protein, one of which (G82R) is linked to increased heparin binding efficiency (38, 39) and attenuated virulence in mice and humans (39, 76, 77). Serial dilutions of viable virus were adsorbed to ELISA plates coated with either heparin or CS, and bound virus was quantified. We calculated a relative binding strength (RBS) for the binding of each strain to heparin and CS, where the RBS values refer to the relative concentration of virus at which 50% of GAG binding sites are occupied.

**TABLE 1:** CHIKV strains used

Clade	Strain	Isolation	Passage history
Asian	Attenuated, 181/25	Tissue culture passage of strain AF15561	11 in GMK cells, 18 in MRC-5 cells
ECSA	Sri Lanka, SL15649	Human patient in Sri Lanka (2006)	3 in Vero cells
Asian	Caribbean, H20235	Human patient in St. Martin (2013)	3 in Vero cells
West African	Senegal, 37997	Mosquito in Senegal (1983)	1 in AP-61 cells, 2 in Vero cells

**FIG 3:**
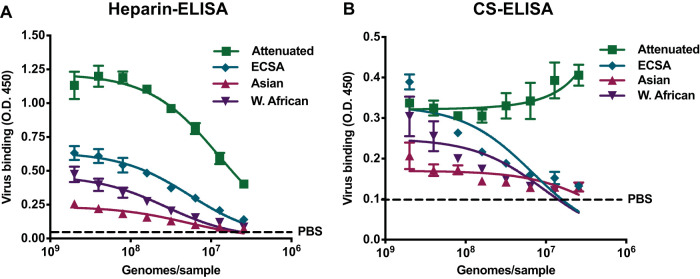
CHIKV strains bind directly to heparin and chondroitin sulfate. Serial dilutions of each CHIKV strain, quantified by genome number, were adsorbed to wells of avidin-coated ELISA plates bound with biotinylated heparin (A) or biotinylated CS (B). PBS was adsorbed to wells coated with heparin and CS as a negative control. Following washes to remove unbound virus, virus binding was detected using a mouse monoclonal anti-CHIKV E2 antibody (CHK-187), secondary goat anti-mouse HRP-conjugated antibody, and TMB substrate. Absorbance was measured at 450 nm for duplicate wells from three independent experiments. The dashed line indicates background levels of binding, as determined using PBS control wells. Error bars indicate SEM. Data were fit using a nonlinear regression curve.

As expected, the attenuated 181/25 strain displayed the highest-avidity binding to heparin (Fig. 3A) and had the lowest RBS value of 7.9 × 10^6^ genomes (Table 2). The other strains tested also bound to heparin in a dose-dependent manner (Fig. 3A). The second-highest heparin binding signals were detected for the ECSA strain, with an RBS value of 1.8 × 10^7^ genomes, followed by moderate binding for the Asian and West African strains (Fig. 3A). The RBS value for heparin binding for the Asian strain was 2 × 10^7^ genomes, and that for the West African strain was 3.6 × 10^7^ genomes (Table 2). In addition, all strains except the attenuated 181/25 strain bound to CS in a dose-dependent manner (Fig. 3B). For this reason, an RBS value for 181/25 binding to CS could not be calculated (Table 2). A similar preference for HS binding relative to CS binding by 181/25 is observed during *in vitro* binding and infection of mutant Chinese hamster ovary cells (38). Similar to heparin binding, the highest binding signals to CS were detected for the ECSA strain, followed by moderate binding for the Asian and West African strains (Fig. 3B). The RBS values for CS binding were 1.4 × 10^7^ genomes for the ECSA strain, 2 × 10^6^ genomes for the Asian strain, and 10^7^ genomes for the West African strain (Table 2). Notably, binding signals were generally lower in the CS binding assays than in the heparin binding assays (Fig. 3). Collectively, these data indicate that CHIKV strains from each clade directly bind *in vitro* to heparin and, to a lesser degree, CS, validating the microarray results that used CHIKV strain 37997 VLPs. These data also demonstrate strain-specific differences in GAG binding, with the ECSA strain binding to heparin and CS with the highest avidity and the Asian strain binding to heparin and CS with the lowest avidity.

**TABLE 2:** CHIKV binding to heparin and CS

Virus	RBS^*a*^			
	Heparin		CS	
	Mean	95% confidence interval	Mean	95% confidence interval
Attenuated	7.9 × 10^6^	6.13 × 10^6^–1.0 × 10^7^	ND^*b*^	ND
ECSA	1.8 × 10^7^	1.3 × 10^7^–2.5 × 10^7^	1.4 × 10^7^	8.8 × 10^6^–2.4 × 10^7^
Asian	2.0 × 10^7^	1.4 × 10^7^–2.9 × 10^7^	2.0 × 10^6^	5.9 × 10^5^–4.4 × 10^6^
West African	3.6 × 10^7^	2.2 × 10^7^–5.9 × 10^7^	1.0 × 10^7^	4.3 × 10^6^–2.5 × 10^7^

a RBS values represent the number of virus genomes at which 50% of GAG binding sites are occupied.

b ND, not determined.

### Enzymatic removal of cell surface HS reduces CHIKV binding and infection.

**FIG 4:**
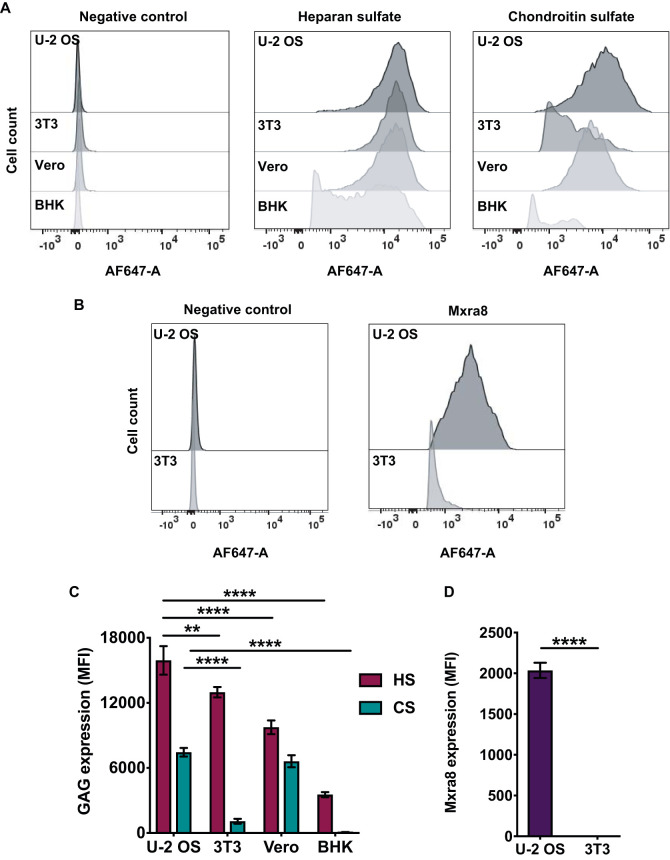
U-2 OS cells express relatively high levels of HS, CS, and Mxra8. U-2 OS, 3T3, Vero, and BHK cells were stained with antibodies specific for HS, CS, or Mxra8, followed by Alexa-647 antibody. Cells were fixed with 4% PFA, and median fluorescence intensity (MFI) was quantified using flow cytometry. (A and B) Representative flow cytometric plots; (C and D) quantification of GAG and Mxra8 profiles for triplicate wells from three independent experiments. Data were normalized to secondary-antibody-only negative controls. Error bars indicate SEM. *P* values were determined by two-way ANOVA followed by Tukey’s multiple-comparison test (**, *P < *0.01; ****, *P < *0.0001).

**FIG 5:**
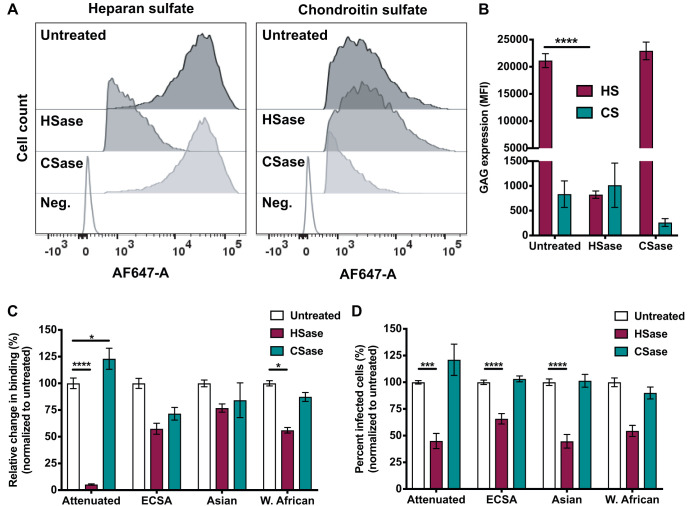
Enzymatic cleavage of HS reduces binding and infection of CHIKV. U-2 OS cells were treated with a combination of heparinases (HSase I, II, and III) or chondroitin sulfatases (CSase ABC) at a final concentration of 2 mIU/ml. (A and B) Cells were stained with antibodies specific for HS or CS, followed by Alexa-647 antibody. Cells were fixed with 4% PFA, and MFI was quantified using flow cytometry. (A) Representative flow cytometric plots; (B) quantification of GAG profiles for duplicate wells from three independent experiments. Data were normalized to secondary-antibody-only negative controls. (C) U-2 OS cells were adsorbed with 10^8^ genomes of the CHIKV strains shown per sample at 4°C for 2 h and washed three times to remove unbound virus. Total RNA was purified using TRIzol, and CHIKV RNA was quantified using RT-qPCR. (D) U-2 OS cells were adsorbed with the attenuated CHIKV strain (181/25) at an MOI of 1 PFU/cell and with the ECSA (SL15649), Asian (H20235), and West African (37997) strains at an MOI of 5 PFU/cell. Cells were fixed with methanol at 18 h postadsorption, and the percentage of infected cells was determined using an immunofluorescence FFU assay. (C and D) Data were normalized to untreated controls. Results are expressed as mean percentage of binding in triplicate wells from three independent experiments (C) or mean percentage of infected cells for four fields of view per well in triplicate wells from three independent experiments (D). (B to D) Error bars indicate SEM. *P* values were determined by two-way ANOVA followed by Tukey’s multiple-comparison test (B) or one-way ANOVA followed by Tukey’s multiple-comparison test (C and D) (*, *P < *0.05; ***, *P < *0.001; ****, *P < *0.0001).

The results obtained thus far demonstrate that multiple CHIKV strains bind GAGs *in vitro*. To determine whether CHIKV-GAG interactions contribute to binding and infection of cells, we treated human osteosarcoma (U-2 OS) cells with a combination of heparinases (HSase) or chondroitinases (CSase) and assessed the cells for GAG expression, virus binding, and virus infectivity. U-2 OS cells were chosen for these experiments because they express higher levels of HS and CS than other cell types commonly used to study CHIKV replication, such as mouse 3T3 fibroblasts, baby hamster kidney (BHK) fibroblasts, and African green monkey kidney epithelial (Vero-81) cells (Fig. 4A and C). U-2 OS cells also express relatively high levels of Mxra8 (Fig. 4B and D), an entry receptor for CHIKV and other arthritogenic alphaviruses (31). Treatment with HSase I, II, or III or CSase ABC specifically and efficiently reduced levels of cell surface HS and CS, respectively (Fig. 5A and B). Following GAG cleavage, Mxra8 expression did not change (data not shown). HS was required for efficient cell binding, as cleavage of HS reduced binding for all CHIKV strains studied (Fig. 5C). As expected, binding of the attenuated 181/25 strain, which has enhanced HS binding capacity (38, 76), was reduced by 95% following HS cleavage (Fig. 5C). Binding of the mosquito and clinical CHIKV strains was reduced by 23% to 44% following HS cleavage (Fig. 5C). Cleavage of CS decreased binding of some CHIKV strains, with that of the ECSA strain reduced by 29%, a reduction greater than that observed for the other strains (Fig. 5C). Additionally, cleavage of HS diminished infectivity of all CHIKV strains by 34% to 55% (Fig. 5D). Cleavage of CS did not affect infectivity (Fig. 5D), suggesting an importance of HS, but not CS, for CHIKV infection of U-2 OS cells. These data indicate that all strains tested depend on HS to bind to cells, while some strains also depend on CS for efficient cell attachment. Thus, efficient infection of U-2 OS cells requires HS binding.

### Genetic ablation of GAG biosynthesis reduces CHIKV binding and infection.

**FIG 6:**
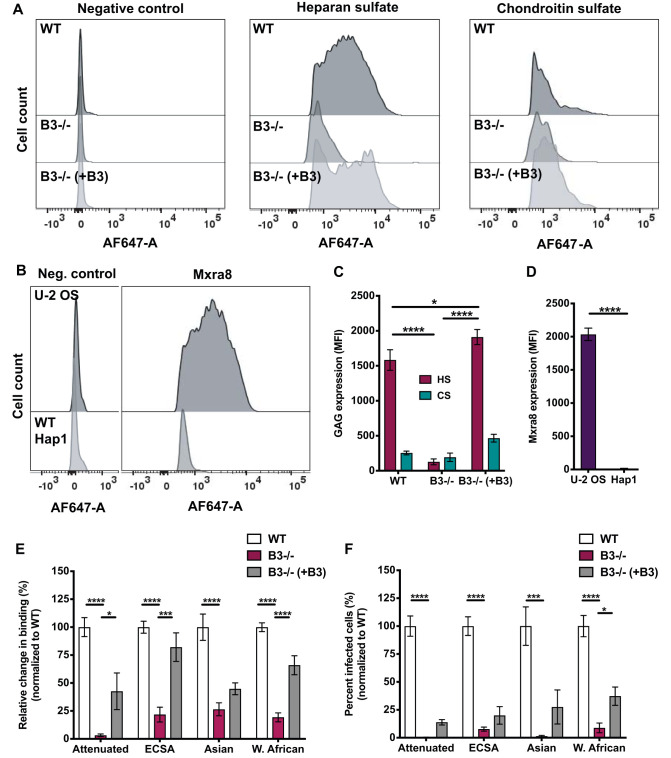
Genetic disruption of GAG biosynthesis reduces CHIKV binding and infection. (A to D) WT, *B3GAT3*^−/−^, and complemented *B3GAT3*^−/−^ HapI cells were stained with antibodies specific for HS, CS, or Mxra8, followed by Alexa-647 antibody. Cells were fixed with 4% PFA, and MFI was quantified using flow cytometry. (A and B) Representative flow cytometric plots; (C and D) quantification of GAG and Mxra8 profiles for triplicate wells from three independent experiments. Data were normalized to secondary-antibody-only negative controls. (E) WT, *B3GAT3*^−/−^, and complemented *B3GAT3*^−/−^ HapI cells were adsorbed with 10^8^ genomes of the virus strains shown per sample at 4°C for 2 h and washed three times to remove unbound virus. Total RNA was purified using TRIzol, and CHIKV RNA was quantified using RT-qPCR. (F) WT, *B3GAT3*^−/−^, and complemented *B3GAT3*^−/−^ HapI cells were adsorbed with the attenuated CHIKV strain (181/25) at an MOI of 2.5 PFU/cell and with the ECSA (SL15649), Asian (H20235), and West African (37997) strains at an MOI of 10 PFU/cell. Cells were fixed with methanol at 18 h postinfection, and the percentage of infected cells was determined using an immunofluorescence FFU assay. (E and F) Data were normalized to WT cells. Results are expressed as mean percentage of binding in triplicate wells from three independent experiments (E) and mean percentage of infected cells for four fields of view per well in triplicate wells from two independent experiments (F). (C to F) Error bars indicate SEM. *P* values were determined by two-way ANOVA followed by Tukey’s multiple-comparison test (C), the two-tailed Student *t* test (D), or one-way ANOVA followed by Tukey’s multiple-comparison test (E and F) (*, *P < *0.05; ***, *P < *0.001; ****, *P < *0.0001).

To investigate the requirement of HS for efficient CHIKV cell binding and infection when Mxra8 and CS are absent, we used human haploid HapI cells. Wild-type (WT) HapI cells abundantly express HS and have low to no expression of CS and Mxra8 (Fig. 6A to D). These features make HapI cells suitable for studies to determine whether HS is required for CHIKV binding and infection. Due to their haploid nature, HapI cells also are more amenable to genetic alteration. We used *B3GAT3*^−/−^ HapI cells, engineered using CRISPR-Cas9 technology (41), which have a targeted disruption of the *B3GAT3* gene, which encodes beta-1,3-glucuronyltransferase 3 (B3GAT3). B3GAT3 catalyzes the transfer of glucuronic acid to galactose, which is a required step in the biosynthesis of heparin, HS, and CS/DS (54). Compared with WT HapI cells, *B3GAT3*^−/−^ cells exhibit diminished GAG expression (Fig. 6A and C). However, neither WT HapI cells nor B3GAT3^−/−^ cells express Mxra8 (Fig. 6B and D and data not shown). *B3GAT3^−/−^* cells complemented with a B3GAT3-expressing plasmid display GAG expression comparable to WT levels (Fig. 6A and C). WT, *B3GAT3*^−/−^, and complemented *B3GAT3*^−/−^ cells were tested for CHIKV binding and infection. Binding to *B3GAT3*^−/−^ cells of all CHIKV strains tested was reduced by 74% to 97% compared with binding to WT cells, and complementation of the *B3GAT3*^−/−^ cells restored binding by 43% to 82% (Fig. 6E). Infection of *B3GAT3*^−/−^ cells by all CHIKV strains tested was diminished by 92% to 100% relative to that of WT cells (Fig. 6F). Complementation of *B3GAT3*^−/−^ cells with *B3GAT3* partially restored infection (Fig. 6F). The lack of full restoration of binding and infection to WT levels after complementation of *B3GAT3*^−/−^ cells may be due to differences in HS expressed by WT and complemented *B3GAT3*^−/−^ cells (Fig. 6A). Overall, these data indicate that CHIKV requires HS for binding to and infection of HapI cells and emphasize the importance of HS as a CHIKV attachment factor when other ligands such as CS or Mxra8 are absent.

## DISCUSSION

The specific glycans used by different strains of CHIKV as attachment factors are not well understood. In this study, we found that sulfated GAGs are the glycans preferentially bound by CHIKV. The strongest binding occurred with HS and heparin, followed by CS. All human- and mosquito-isolated CHIKV strains tested directly bound to heparin and CS. HS was required for efficient binding and infection of U-2 OS and HapI cells, while CS was required by only some strains to efficiently attach to U-2 OS cells. Moreover, the requirement of GAGs for CHIKV binding and infection inversely correlated with levels of Mxra8 receptor expression. Collectively, these data suggest that HS and, to a lesser extent, CS function as attachment factors for several CHIKV strains.

CHIKV displays broad cell, tissue, and species tropism (8, 78), which may correlate with the attachment factors or entry receptors used by the virus. Previous studies, as well as this work, identified sulfated GAGs as CHIKV attachment factors (38, 40, 41) (Fig. 5 and 6). These glycans are ubiquitously expressed in humans and mosquitoes (54–56), including the specific cells and tissues that CHIKV infects. Many pathogenic viruses, including viruses in the alphavirus family (42–45, 52, 79) as well as other virus families (46–51, 53, 80–83), bind GAG attachment factors to attach to cells. For example, enterovirus 71 (EV-71), which displays broad tissue tropism (84) like CHIKV, specifically binds HS as an attachment factor (53). An alphavirus, eastern equine encephalitis virus, also binds HS attachment factors (43). Strains of both EV-71 and eastern equine encephalitis virus with enhanced HS binding capacity display broadened tissue tropism and enhanced virulence (42, 43, 85). Thus, GAG attachment factor binding can influence viral tropism and virulence.

Although GAGs are CHIKV attachment factors, the specific GAG sequences required for CHIKV binding had not been defined. GAG types and sequences vary in different cells, tissues, and organisms, and the interactions between GAGs and proteins are often mediated by the structural characteristics of GAG chains. GAG types differ in their composition of repeating disaccharide units, which can facilitate specific interactions with chemokines, growth factors, enzymes, and viral proteins (86–88). The glycan microarray analyses we conducted identified sulfated GAGs as the primary glycan type bound by chikungunya VLPs (Fig. 1), with HS and heparin most strongly bound (Fig. 2). Similarly, binding signals were generally lower in the CS ELISAs than in the heparin ELISAs, suggesting a preference of CHIKV for binding to heparin (Fig. 3). On the glycan microarrays, some weak binding to non-GAG glycans also was detected, which may prompt further investigation into these CHIKV-glycan interactions. Interestingly, the iduronic acid-containing GAGs, HS, heparin, and CS-B (DS), which are abundantly expressed in cells and tissues infected by CHIKV (54–56, 60–64), had the highest binding signals with VLPs relative to those of other GAGs tested (Fig. 2). This is reminiscent of the GAG binding properties of respiratory syncytial virus (RSV), which requires iduronic acid-containing GAGs for *in vitro* infection (51).

GAG oligosaccharide chain length is another important structural characteristic that influences binding to many ligands, including chemokines, growth factors, tau aggregates, and viral proteins (57, 89). We found that longer, sulfated GAGs are generally bound more efficiently by CHIKV (Fig. 1 and 2). VLPs bound more efficiently to longer rather than shorter chains of almost every GAG type (Fig. 2). The requirement of longer GAG chains for virus binding has been observed for many viruses (82, 83, 90–92). For example, RSV requires heparin with a minimum 10-mer chain for efficient binding (82), and Zika virus preferentially binds 8- to 18-mer heparin chains (83). Further investigation is required to determine the optimum chain length required for each GAG type to promote binding of different CHIKV strains.

Sulfation modifications along the GAG chain also regulate ligand binding (93). Our studies indicate that the degree of sulfation is an important factor in CHIKV-GAG binding, which is consistent with previous findings demonstrating that N-sulfation of HS chains is required for CHIKV infection *in vitro* (41). VLPs bound to all sulfated GAGs and dextran sulfate but not to hyaluronan or dextran, which are unsulfated glycans (Fig. 2). GAG sulfation also influences the binding of several other viruses (80–82, 94–96). In fact, specific sulfation modifications on HS chains are important for virus-GAG interactions, such as 3-O sulfation for herpes simplex virus 1 (95, 96) and N-sulfation for RSV (82). Although we found that sulfation of GAG chains is required for CHIKV binding, the specific sulfation patterns necessary for CHIKV engagement remain unknown. Given that expression of many sulfation-modifying enzymes is tissue specific (58, 97, 98), identifying the specific modifications necessary for CHIKV binding could enhance our understanding of its tropism and help define more specific cell attachment inhibitors. Collectively, our glycan microarray analyses suggest that CHIKV most efficiently binds longer, sulfated GAGs, with a preference for HS and heparin. As GAG mimetics are a possible therapeutic for alphavirus and flavivirus disease (99–102), understanding the unique GAG sequences required for efficient CHIKV binding may foster development of new classes of GAG-based antiviral agents.

In addition to identifying specific GAGs bound by CHIKV, we evaluated strain-specific differences in GAG attachment during infection of cells. Strain differences in CHIKV tropism and virulence have been observed (39, 103, 104). Therefore, it is important to know whether CHIKV strains also differ in attachment factor binding, which often is a determinant of tropism and virulence. Several cell culture-adapted alphaviruses (105–107), including CHIKV strain 181/25 (38, 39, 66), bind to GAGs. GAG binding was previously thought to be attributable to a cell culture adaptation that was dispensable for infection by naturally circulating alphavirus strains. However, evidence has accumulated supporting a role for GAG binding by clinically relevant, non-culture-adapted alphaviruses (38, 39, 43–45, 52). Using viruses that were minimally passaged in culture, we discovered that the ECSA strain bound most efficiently to heparin and CS (Fig. 3) and was the only strain that required both HS and CS expression to efficiently bind to U-2 OS cells (Fig. 5C). In contrast, the Asian strain bound less efficiently to GAGs (Fig. 3), and virus binding was least affected by HS cleavage on U-2 OS cells (Fig. 5C) and the absence of HS on HapI *B3GAT3*^−/−^ cells (Fig. 6E). These results parallel the requirement for Mxra8 utilization for infection of fibroblasts *in vitro*, with Asian and ECSA strains showing full and partial Mxra8 dependence for infection, respectively (31). Similarities between the strains also were observed. All CHIKV strains tested required HS to efficiently bind and infect U-2 OS and HapI cells (Fig. 5 and 6). Interestingly, following HS cleavage of U-2 OS cells, residual CHIKV binding (56 to 77%) and infection (44 to 66%) were observed (Fig. 5C and D). However, residual CHIKV binding to (19 to 26%) and infection of (1 to 9%) HapI *B3GAT3*^−/−^ cells were significantly less (Fig. 6E and F). The low expression of Mxra8 and CS on HapI cells compared to U-2 OS cells may influence the observed differences in residual binding and infection. These data suggest that although HS is required for efficient CHIKV binding and infection, the magnitude of the requirement is inversely correlated with the abundance of entry receptor expression. Additionally, the residual binding to and infection of HapI *B3GAT3*^−/−^ cells, which express little to no GAGs or Mxra8, suggest the presence of an unidentified cell surface molecule engaged by CHIKV or a route of viral entry other than receptor-mediated endocytosis.

Our studies contribute to an understanding of the interactions between CHIKV and the cell surface molecules that promote virus attachment. We have identified specific GAG types to which CHIKV binds as well as differences in the binding efficiency of CHIKV to specific GAGs. Using clinically relevant CHIKV strains, we discovered strain-specific differences in GAG binding and the requirement of GAGs for attachment and infection of cultured cells. Our data demonstrate that multiple strains of CHIKV bind HS and CS as attachment factors, likely promoting initial cell attachment and allowing the virus to concentrate at the cell surface before engaging entry receptors. CHIKV interactions with widely expressed GAGs may contribute to the broad cell, tissue, and species tropism observed for CHIKV. Overall, the findings reported here define critical interactions between CHIKV and GAG attachment factors and improve understanding of the multistep process of cell attachment for CHIKV.

## MATERIALS AND METHODS

### Cells.

Baby hamster kidney cells (BHK-21; ATCC CCL-10) were maintained in alpha minimal essential medium (αMEM) (Gibco) supplemented to contain 10% fetal bovine serum (FBS) (VWR) and 10% tryptose phosphate (Sigma). Vero 81 cells (ATCC CCL-81) were maintained in αMEM supplemented to contain 5% FBS. Human osteosarcoma cells (U-2 OS; ATCC HTB-96) were maintained in McCoy’s 5A medium (Gibco) supplemented to contain 10% FBS. Culture media for BHK-21, Vero-81, and U-2 OS cells also were supplemented with 0.29 mg/ml l-glutamine (Gibco), 100 U/ml penicillin (Gibco), 100 μg/ml streptomycin (Gibco), and 25 ng/ml amphotericin B (Sigma). WT and *B3GAT3^−/−^* human HapI cells (41) were provided by Yusuke Maeda (Osaka University) and Atsushi Tanaka (Thailand-Japan RCC-ERI). HapI cells were maintained in Iscove’s modified Dulbecco’s medium (Gibco) supplemented to contain 10% FBS, 100 U/ml penicillin, and 100 μg/ml streptomycin. All cells were cultivated at 37°C in an atmosphere of 5% CO_2_.

### VLPs and viruses.

Chikungunya VLPs of the 37997 strain were prepared by Emergent BioSolutions as described previously (108). Suspension-adapted, serum-free human embryonic kidney 293 cells were transfected with an expression plasmid containing strain 37997 structural genes. Supernatants were collected and clarified by centrifugation. VLPs were purified using chromatography and sterile filtration, suspended in 10 mM potassium phosphate, 218 mM sucrose, and 25 mM sodium citrate, and stored at −80°C prior to use.

Virus stocks were recovered from infectious cDNA clone plasmids for each CHIKV strain (Table 1), including 181/25 (67, 68), SL15649 (29), H20235 (75), and 37997 (70). Plasmids were linearized with NotI-HF (NEB) and transcribed *in vitro* using an mMessage mMachine SP6 transcription kit (Ambion). BHK-21 cells (1.19 × 10^7^ cells) were electroporated with *in vitro*-transcribed RNA using a Gene Pulser Xcell electroporator (Bio-Rad) and the square wave protocol with 2 pulses at 1,000 V for 2.5 ms with 5 s between each pulse. Cells were incubated at 37°C for 48 h. Supernatants were collected and clarified by centrifugation at 1,500 × *g* at 4°C for 10 min to remove cell debris. The remaining supernatant was added to a 20% sucrose cushion in TNE buffer (phosphate-buffered saline without calcium or magnesium [PBS^−/−^] supplemented to contain 50 mM Tris-HCl [pH 7.2], 0.1 M NaCl, and 1 mM EDTA) and centrifuged at ∼115,000 × *g* for ∼16 h in a Beckman 32Ti rotor. Pellets containing virus were resuspended in virus dilution buffer (VDB) (RPMI medium supplemented to contain 20 mM HEPES [Gibco] and 1% FBS), aliquoted, and stored at −80°C. Titers of virus stocks were determined by plaque assay. Genome copy numbers of virus stocks were determined by reverse transcription-quantitative PCR (RT-qPCR).

### Viral plaque assays.

Confluent monolayers of Vero-81 cells were adsorbed with serial dilutions (10-fold) of virus stocks in VDB at 37°C for 1 h. Cells were overlaid with 0.5% immunodiffusion agarose (VWR) in αMEM supplemented to contain 10% FBS, 10% tryptose phosphate, 100 U/ml penicillin, and 100 μg/ml streptomycin. Cells were incubated at 37°C for ∼48 h. Plaques were visualized following staining with neutral red (Sigma) at 37°C for 4 to 6 h. Plaques were enumerated in duplicate and averaged to calculate PFU.

### Viral RT-qPCR.

Viral RNA was extracted from 10 μl of purified virus stocks using 490 μl TRIzol reagent (Thermo Fisher Scientific), purified using the PureLink RNA minikit (Invitrogen), and eluted into a final volume of 100 μl. Viral genomes were quantified using the qScript XLT one-step RT-qPCR ToughMix kit (Quanta Biosciences). Reactions were conducted in 20 μl, containing 5 μl viral RNA, 500 nM forward primer (5′-AGACCAGTCGACGTGTTGTAC-3′), 500 nM reverse primer (5′-GTGCGCATTTTGCCTTCGTA-3′), and 250 nM fluorogenic probe (5′-/56-FAM/ATCTGCACC/ZEN/CAAGTGTACCA/3IABkFQ/-3′), targeting an amplicon in the nonstructural protein 2 (nsp2)-coding region. Standard curves for each virus strain were prepared using *in vitro*-transcribed viral RNA. RT-qPCR was conducted using a ViiA 7 real-time PCR system (Thermo Fisher Scientific) under the following conditions: 50°C for 10 min, 95°C for 10 min, 40 cycles of 95°C for 15 s, and 60°C for 60 s, with data acquisition in the FAM channel during the 60°C step. RNA concentrations were determined by comparing the threshold cycle (*C_T_*) values of each sample to an appropriate standard curve. RT-qPCR to determine genome copy numbers of virus stocks (genomes per milliliter) were conducted in triplicate.

### Glycan microarrays

The binding specificities of the chikungunya virus 37997 VLPs were analyzed using a neoglycolipid (NGL)-based microarray system (109). Two types of microarrays were used: (i) glycan microarrays composed of 672 sequence-defined lipid-linked mammalian and nonmammalian glycans as described previously (110) and (ii) GAG-focused microarrays composed of NGL probes of 13 size-defined glycosaminoglycan (GAG) oligosaccharides and two non-GAG polysaccharide controls. The glycan probes and sequences used in the glycan microarrays are provided in Table S1 in the supplemental material. The glycan probes and sequences used in the GAG-focused arrays are provided in Fig. 2B. Information about the preparation of the glycan probes and construction of the microarrays is presented in Table S3 in the supplemental material in accordance with the MIRAGE (Minimum Information Required for a Glycomics Experiment) guidelines for reporting of glycan microarray-based data (111).

Multiple analyses were conducted with the chikungunya VLPs and anti-CHIKV antibodies (Table S3). Slides were blocked at room temperature for 1 h with HBS buffer (10 mM HEPES at pH 7.4 with 150 mM NaCl and 5 mM CaCl_2_) supplemented to contain 0.02% (wt/vol) casein (Pierce) and 1% (wt/vol) bovine serum albumin (BSA) (Sigma). Microarrays were overlaid with VLP solution (50 μg/ml was used in most analyses) at room temperature for 1.5 h and fixed with 4% paraformaldehyde (PFA) diluted in high-pressure liquid chromatography (HPLC)-grade water at 4°C for 30 min. VLP binding was detected following incubation with anti-CHIKV E2 antibody (CHK-152 [112]; 1:300) or ascites fluid (ATCC VR-1241AF; 1:300) at room temperature for 1 h, biotinylated goat anti-mouse IgG (Sigma; 2 μg/ml) at room temperature for 1 h, and Alexa Fluor 647-labeled streptavidin (Molecular Probes;1 μg/ml) at room temperature for 30 min. Imaging and data analysis are described in the supplementary MIRAGE document (Table S3).

### ELISAs and RBS calculations.

Pierce NeutrAvidin-coated ELISA plates (Thermo Fisher Scientific 15123B) were adsorbed with 4 ng/μl of heparin conjugated to biotin (Creative PEGWorks HP-207) or 15 ng/μl of chondroitin sulfate conjugated to biotin (Creative PEGWorks CS-106; mixture of CS-A, CS-B, and CS-C) at room temperature for 2 h. Wells were washed three times with wash buffer (PBS^−/−^ supplemented to contain 0.05% Tween 20). ELISA plates were adsorbed with serial dilutions (1:2) of virus in VDB at room temperature for 1 h. As a negative control, PBS^−/−^ was adsorbed to ELISA plates coated with heparin and CS. Wells were washed with wash buffer three times to remove unbound virus. Bound virus was detected following incubation with a mouse monoclonal anti-CHIKV E2 antibody (CHK-187 [112]; 1:1,000) at room temperature for 1 h, a horseradish peroxidase-conjugated goat anti-mouse Ig (SouthernBiotech 2040-05) at room temperature for 1 h, and tetramethylbenzidine (TMB) substrate (Thermo Fisher Scientific) for up to 5 min. Absorbance at 450 nm was quantified using a Synergy H1 microplate reader (BioTek). Data were used to prepare a nonlinear regression curve assuming one-site specific binding, and relative binding strength (RBS) values were calculated for each virus. RBS values refer to the number of genomes of virus at which 50% of GAG binding sites are occupied.

### Cell surface glycan and protein expression.

Cells were detached from tissue culture flasks using CellStripper dissociation reagent (Corning), quenched with PBS with calcium and magnesium (PBS^+/+^) supplemented to contain 2% FBS, and centrifuged at 1,500 × *g* at 4°C for 5 min. Cells (5 × 10^5^ cells per sample) were stained with human anti-HS (1:750; Amsbio 370255-S), human anti-CS (1:750; Sigma C8035), human anti-Mxra8 (1 μg/ml; MBL International W040-3), or mouse anti-Mxra8 (1 μg/ml; 4E7.D10 [31]) antibodies at 4°C for 1 h. Cells were incubated with Alexa Fluor 647 antibody (1:1,000; Thermo Fischer Scientific) at 4°C for 1 h. Samples were washed twice with VDB between incubations. Samples were fixed with 1% PFA at 4°C for 5 min and analyzed by flow cytometry (LSRII flow cytometer; BD Biosciences). Binding events were gated using secondary-antibody-only control samples as the no-binding controls, and median fluorescent intensity (MFI) was determined using FlowJo V10 software.

### Virus binding to cells.

Cells were detached from tissue culture flasks using CellStripper dissociation reagent, quenched with PBS^+/+^ supplemented to contain 2% FBS, and centrifuged at 1,500 × *g* at 4°C for 5 min. Cells (5 × 10^5^ cells per sample) were adsorbed with virus at 10^8^ genomes per sample at 4°C for 2 h and washed three times with VDB. Cells were centrifuged at 1,500 × *g* for 5 min, and pellets were resuspended in 750 μl of TRIzol. RNA was purified, and viral genomes per sample were quantified using RT-qPCR.

### Focus-forming unit (FFU) assays.

Virus was adsorbed to monolayers of U-2 OS or HapI cells at the multiplicities of infection (MOIs) indicated in the figure legends. Following incubation at 37°C for 1 h, the inoculum was removed, and cells were incubated at 37°C for 18 h in medium supplemented to contain 20 mM NH_4_Cl. Cells were fixed with ice-cold methanol for 30 min and washed three times with PBS^−/−^. Blocking buffer (PBS^+/+^ supplemented to contain 5% FBS and 0.1% TX-100) was added to the plate at room temperature and left for 1 h. Cells were stained with anti-CHIKV ascites fluid (1:1,500; ATCC VR-1241AF) at room temperature for 1 h and with goat anti-mouse Alexa Fluor 488 IgG (1:1,000; Invitrogen A11029) with 4′,6-diamidino-2-phenylindole (DAPI) (1:1,000; Thermo Fisher Scientific) at room temperature for 1 h. Cells were washed with PBS^−/−^ three times at room temperature for 5 min per wash between each staining step. Infectivity was quantified by indirect immunofluorescence using the Lionheart FX automated microscope and Gen5 software (BioTek).

### GAG cleavage assays.

U-2 OS cells were adsorbed with heparinases (HSase I, II, and III; Sigma H2519, H6512, and H8891, respectively) or chondroitinases (CSase ABC; Sigma C3667) at a final concentration of 2 mIU/ml diluted in digestion buffer (MilliQ water supplemented to contain 20 mM HEPES [pH 7.5], 150 mM NaCl, 4 mM CaCl_2_, and 0.1% BSA) at 37°C for 1 h. Cells were washed with PBS^−/−^ three times. Cell surface GAG expression was quantified by flow cytometry, virus binding by RT-qPCR, and virus infectivity by FFU.

### Transient complementation of KO cells.

HapI *B3GAT3^−/−^* cells were transfected with pcDNA3.1(+)-N-eGFP containing human *B3GAT3* (GenScript OHu21110C) using Lipofectamine 3000 (Thermo Fisher Scientific L3000015) at a 3:1 transfection reagent-to-DNA ratio. Medium was changed at 24 h posttransfection. At 36 h posttransfection, cell surface GAG expression was quantified by flow cytometry, virus binding by RT-qPCR, and virus infectivity by FFU assay.

### Statistical analysis.

Statistical tests were conducted using GraphPad PRISM 7 software. *P* values of less than 0.05 were considered to be statistically significant. Descriptions of the specific statistical tests are provided in the figure legends.

### Biosafety.

All studies using VLPs were conducted using biosafety level 2 conditions, and all studies using viable virus were conducted in a certified biosafety level 3 facility. Protocols used were approved by the University of Pittsburgh Department of Environment, Health, and Safety and the University of Pittsburgh Institutional Biosafety Committee.

## Supplementary Material

Supplemental file 1

Supplemental file 2

Supplemental file 3

## References

[1] Mercer J, Schelhaas M, Helenius A. 2010. Virus entry by endocytosis. Annu Rev Biochem 79:803–833. doi:10.1146/annurev-biochem-060208-104626. 20196649

[2] Marsh M, Helenius A. 2006. Virus entry: open sesame. Cell 124:729–740. doi:10.1016/j.cell.2006.02.007. 16497584 PMC7112260

[3] Casasnovas JM. 2013. Virus-receptor interactions and receptor-mediated virus entry into host cells. Subcell Biochem 68:441–466. doi:10.1007/978-94-007-6552-8_15. 23737061 PMC7122110

[4] Halstead SB. 2015. Reappearance of chikungunya, formerly called dengue, in the Americas. Emerg Infect Dis 21:557–561. doi:10.3201/eid2104.141723. 25816211 PMC4378492

[5] Thiberville S-D, Moyen N, Dupuis-Maguiraga L, Nougairede A, Gould EA, Roques P, de Lamballerie X. 2013. Chikungunya fever: epidemiology, clinical syndrome, pathogenesis and therapy. Antiviral Res 99:345–370. doi:10.1016/j.antiviral.2013.06.009. 23811281 PMC7114207

[6] Powers AM, Logue CH. 2007. Changing patterns of chikungunya virus: re-emergence of a zoonotic arbovirus. J Gen Virol 88:2363–2377. doi:10.1099/vir.0.82858-0. 17698645

[7] Staples JE, Breiman RF, Powers AM. 2009. Chikungunya fever: an epidemiological review of a re-emerging infectious disease. Clin Infect Dis 49:942–948. doi:10.1086/605496. 19663604

[8] Schwartz O, Albert ML. 2010. Biology and pathogenesis of chikungunya virus. Nat Rev Microbiol 8:491–500. doi:10.1038/nrmicro2368. 20551973

[9] Gibney KB, Fischer M, Prince HE, Kramer LD, St George K, Kosoy OL, Laven JJ, Staples JE. 2011. Chikungunya fever in the United States: a fifteen year review of cases. Clin Infect Dis 52:e121-6–e126. doi:10.1093/cid/ciq214. 21242326

[10] PAHO. 2018. Geographic spread of chikungunya in the Americas December 2013–December 2017. PAHO, Washington, DC.

[11] Silva LA, Dermody TS. 2017. Chikungunya virus: epidemiology, replication, disease mechanisms, and prospective intervention strategies. J Clin Invest 127:737–749. doi:10.1172/JCI84417. 28248203 PMC5330729

[12] Arankalle VA, Shrivastava S, Cherian S, Gunjikar RS, Walimbe AM, Jadhav SM, Sudeep AB, Mishra AC. 2007. Genetic divergence of chikungunya viruses in India (1963–2006) with special reference to the 2005–2006 explosive epidemic. J Gen Virol 88:1967–1976. doi:10.1099/vir.0.82714-0. 17554030

[13] Cassadou S, Boucau S, Petit-Sinturel M, Huc P, Leparc-Goffart I, Ledrans M. 2014. Emergence of chikungunya fever on the French side of Saint Martin Island, October to December 2013. Euro Surveill 19:20752. doi:10.2807/1560-7917.ES2014.19.13.20752. 24721536

[14] Dupuis-Maguiraga L, Noret M, Brun S, Le Grand R, Gras G, Roques P. 2012. Chikungunya disease: infection-associated markers from the acute to the chronic phase of arbovirus-induced arthralgia. PLoS Negl Trop Dis 6:e1446. doi:10.1371/journal.pntd.0001446. 22479654 PMC3313943

[15] Ayu SM, Lai LR, Chan YF, Hatim A, Hairi NN, Ayob A, Sam I-C. 2010. Seroprevalence survey of Chikungunya virus in Bagan Panchor, Malaysia. Am J Trop Med Hyg 83:1245–1248. doi:10.4269/ajtmh.2010.10-0279. 21118929 PMC2990039

[16] Borgherini G, Poubeau P, Jossaume A, Gouix A, Cotte L, Michault A, Arvin-Berod C, Paganin F. 2008. Persistent arthralgia associated with chikungunya virus: a study of 88 adult patients on Reunion Island. Clin Infect Dis 47:469–475. doi:10.1086/590003. 18611153

[17] Lemant J, Boisson V, Winer A, Thibault L, André H, Tixier F, Lemercier M, Antok E, Cresta MP, Grivard P, Besnard M, Rollot O, Favier F, Huerre M, Campinos JL, Michault A. 2008. Serious acute chikungunya virus infection requiring intensive care during the Reunion Island outbreak in 2005–2006. Crit Care Med 36:2536–2541. doi:10.1097/CCM.0b013e318183f2d2. 18679124

[18] Schilte C, Staikowsky F, Staikovsky F, Couderc T, Madec Y, Carpentier F, Kassab S, Albert ML, Lecuit M, Michault A. 2013. Chikungunya virus-associated long-term arthralgia: a 36-month prospective longitudinal study. PLoS Negl Trop Dis 7:e2137. doi:10.1371/journal.pntd.0002137. 23556021 PMC3605278

[19] Soumahoro M-K, Boelle P-Y, Gaüzere B-A, Atsou K, Pelat C, Lambert B, La Ruche G, Gastellu-Etchegorry M, Renault P, Sarazin M, Yazdanpanah Y, Flahault A, Malvy D, Hanslik T. 2011. The Chikungunya epidemic on La Réunion Island in 2005–2006: a cost-of-illness study. PLoS Negl Trop Dis 5:e1197. doi:10.1371/journal.pntd.0001197. 21695162 PMC3114750

[20] Diallo D, Sall AA, Buenemann M, Chen R, Faye O, Diagne CT, Faye O, Ba Y, Dia I, Watts D, Weaver SC, Hanley KA, Diallo M. 2012. Landscape ecology of sylvatic chikungunya virus and mosquito vectors in southeastern Senegal. PLoS Negl Trop Dis 6:e1649. doi:10.1371/journal.pntd.0001649. 22720097 PMC3373654

[21] McFarlane M, Arias-Goeta C, Martin E, O'Hara Z, Lulla A, Mousson L, Rainey SM, Misbah S, Schnettler E, Donald CL, Merits A, Kohl A, Failloux A-B. 2014. Characterization of *Aedes aegypti* innate-immune pathways that limit chikungunya virus replication. PLoS Negl Trop Dis 8:e2994. doi:10.1371/journal.pntd.0002994. 25058001 PMC4109886

[22] Sirisena PDNN, Kumar A, Sunil S. 2018. Evaluation of Aedes aegypti (Diptera: Culicidae) life table attributes upon chikungunya virus replication reveals impact on egg-laying pathways. J Med Entomol 55:1580–1587. doi:10.1093/jme/tjy097. 29931258

[23] Sourisseau M, Schilte C, Casartelli N, Trouillet C, Guivel-Benhassine F, Rudnicka D, Sol-Foulon N, Le Roux K, Prevost M-C, Fsihi H, Frenkiel M-P, Blanchet F, Afonso PV, Ceccaldi P-E, Ozden S, Gessain A, Schuffenecker I, Verhasselt B, Zamborlini A, Saïb A, Rey FA, Arenzana-Seisdedos F, Desprès P, Michault A, Albert ML, Schwartz O. 2007. Characterization of reemerging chikungunya virus. PLoS Pathog 3:e89. doi:10.1371/journal.ppat.0030089. 17604450 PMC1904475

[24] Her Z, Malleret B, Chan M, Ong EKS, Wong S-C, Kwek DJC, Tolou H, Lin RTP, Tambyah PA, Rénia L, Ng LFP. 2010. Active infection of human blood monocytes by Chikungunya virus triggers an innate immune response. J Immunol 184:5903–5913. doi:10.4049/jimmunol.0904181. 20404274

[25] Puiprom O, Morales Vargas RE, Potiwat R, Chaichana P, Ikuta K, Ramasoota P, Okabayashi T. 2013. Characterization of chikungunya virus infection of a human keratinocyte cell line: role of mosquito salivary gland protein in suppressing the host immune response. Infect Genet Evol 17:210–215. doi:10.1016/j.meegid.2013.04.005. 23583544

[26] Ozden S, Huerre M, Riviere J-P, Coffey LL, Afonso PV, Mouly V, de Monredon J, Roger J-C, El Amrani M, Yvin J-L, Jaffar M-C, Frenkiel M-P, Sourisseau M, Schwartz O, Butler-Browne G, Desprès P, Gessain A, Ceccaldi P-E. 2007. Human muscle satellite cells as targets of Chikungunya virus infection. PLoS One 2:e527. doi:10.1371/journal.pone.0000527. 17565380 PMC1885285

[27] Couderc T, Chrétien F, Schilte C, Disson O, Brigitte M, Guivel-Benhassine F, Touret Y, Barau G, Cayet N, Schuffenecker I, Desprès P, Arenzana-Seisdedos F, Michault A, Albert ML, Lecuit M. 2008. A mouse model for Chikungunya: young age and inefficient type-I interferon signaling are risk factors for severe disease. PLoS Pathog 4:e29. doi:10.1371/journal.ppat.0040029. 18282093 PMC2242832

[28] Gardner J, Anraku I, Le TT, Larcher T, Major L, Roques P, Schroder WA, Higgs S, Suhrbier A. 2010. Chikungunya virus arthritis in adult wild-type mice. J Virol 84:8021–8032. doi:10.1128/JVI.02603-09. 20519386 PMC2916516

[29] Morrison TE, Oko L, Montgomery SA, Whitmore AC, Lotstein AR, Gunn BM, Elmore SA, Heise MT. 2011. A mouse model of chikungunya virus-induced musculoskeletal inflammatory disease: evidence of arthritis, tenosynovitis, myositis, and persistence. Am J Pathol 178:32–40. doi:10.1016/j.ajpath.2010.11.018. 21224040 PMC3069999

[30] Young AR, Locke MC, Cook LE, Hiller BE, Zhang R, Hedberg ML, Monte KJ, Veis DJ, Diamond MS, Lenschow DJ. 2019. Dermal and muscle fibroblasts and skeletal myofibers survive chikungunya virus infection and harbor persistent RNA. PLoS Pathog 15:e1007993. doi:10.1371/journal.ppat.1007993. 31465513 PMC6715174

[31] Zhang R, Kim AS, Fox JM, Nair S, Basore K, Klimstra WB, Rimkunas R, Fong RH, Lin H, Poddar S, Crowe JE, Doranz BJ, Fremont DH, Diamond MS. 2018. Mxra8 is a receptor for multiple arthritogenic alphaviruses. Nature 557:570–574. doi:10.1038/s41586-018-0121-3. 29769725 PMC5970976

[32] Zhang R, Earnest JT, Kim AS, Winkler ES, Desai P, Adams LJ, Hu G, Bullock C, Gold B, Cherry S, Diamond MS. 2019. Expression of the mxra8 receptor promotes alphavirus infection and pathogenesis in mice and drosophila. Cell Rep 28:2647–2658. doi:10.1016/j.celrep.2019.07.105. 31484075 PMC6745702

[33] Wintachai P, Wikan N, Kuadkitkan A, Jaimipuk T, Ubol S, Pulmanausahakul R, Auewarakul P, Kasinrerk W, Weng W-Y, Panyasrivanit M, Paemanee A, Kittisenachai S, Roytrakul S, Smith DR. 2012. Identification of prohibitin as a Chikungunya virus receptor protein. J Med Virol 84:1757–1770. doi:10.1002/jmv.23403. 22997079

[34] Moller-Tank S, Kondratowicz AS, Davey RA, Rennert PD, Maury W. 2013. Role of the phosphatidylserine receptor TIM-1 in enveloped-virus entry. J Virol 87:8327–8341. doi:10.1128/JVI.01025-13. 23698310 PMC3719829

[35] Fongsaran C, Jirakanwisal K, Kuadkitkan A, Wikan N, Wintachai P, Thepparit C, Ubol S, Phaonakrop N, Roytrakul S, Smith DR. 2014. Involvement of ATP synthase β subunit in chikungunya virus entry into insect cells. Arch Virol 159:3353–3364. doi:10.1007/s00705-014-2210-4. 25168043

[36] Klimstra WB, Nangle EM, Smith MS, Yurochko AD, Ryman KD. 2003. DC-SIGN and L-SIGN can act as attachment receptors for alphaviruses and distinguish between mosquito cell- and mammalian cell-derived viruses. J Virol 77:12022–12032. doi:10.1128/jvi.77.22.12022-12032.2003. 14581539 PMC254289

[37] Carnec X, Meertens L, Dejarnac O, Perera-Lecoin M, Hafirassou ML, Kitaura J, Ramdasi R, Schwartz O, Amara A. 2016. The phosphatidylserine and phosphatidylethanolamine receptor cd300a binds dengue virus and enhances infection. J Virol 90:92–102. doi:10.1128/JVI.01849-15. 26468529 PMC4702537

[38] Silva LA, Khomandiak S, Ashbrook AW, Weller R, Heise MT, Morrison TE, Dermody TS. 2014. A single-amino-acid polymorphism in Chikungunya virus E2 glycoprotein influences glycosaminoglycan utilization. J Virol 88:2385–2397. doi:10.1128/JVI.03116-13. 24371059 PMC3958064

[39] Ashbrook AW, Burrack KS, Silva LA, Montgomery SA, Heise MT, Morrison TE, Dermody TS. 2014. Residue 82 of the Chikungunya virus E2 attachment protein modulates viral dissemination and arthritis in mice. J Virol 88:12180–12192. doi:10.1128/JVI.01672-14. 25142598 PMC4248890

[40] Weber C, Berberich E, von Rhein C, Henß L, Hildt E, Schnierle BS. 2017. Identification of functional determinants in the chikungunya virus E2 protein. PLoS Negl Trop Dis 11:e0005318. doi:10.1371/journal.pntd.0005318. 28114368 PMC5289616

[41] Tanaka A, Tumkosit U, Nakamura S, Motooka D, Kishishita N, Priengprom T, Sa-Ngasang A, Kinoshita T, Takeda N, Maeda Y. 2017. Genome-wide screening uncovers the significance of N-sulfation of heparan sulfate as a host cell factor for chikungunya virus infection. J Virol 91:e00432-17. doi:10.1128/JVI.00432-17. 28404855 PMC5469253

[42] Gardner CL, Choi-Nurvitadhi J, Sun C, Bayer A, Hritz J, Ryman KD, Klimstra WB. 2013. Natural variation in the heparan sulfate binding domain of the eastern equine encephalitis virus E2 glycoprotein alters interactions with cell surfaces and virulence in mice. J Virol 87:8582–8590. doi:10.1128/JVI.00937-13. 23720725 PMC3719831

[43] Gardner CL, Ebel GD, Ryman KD, Klimstra WB. 2011. Heparan sulfate binding by natural eastern equine encephalitis viruses promotes neurovirulence. Proc Natl Acad Sci U S A 108:16026–16031. doi:10.1073/pnas.1110617108. 21896745 PMC3179095

[44] Byrnes AP, Griffin DE. 1998. Binding of Sindbis virus to cell surface heparan sulfate. J Virol 72:7349–7356. doi:10.1128/JVI.72.9.7349-7356.1998. 9696831 PMC109959

[45] Zhang W, Heil M, Kuhn RJ, Baker TS. 2005. Heparin binding sites on Ross River virus revealed by electron cryo-microscopy. Virology 332:511–518. doi:10.1016/j.virol.2004.11.043. 15680416 PMC4152768

[46] WuDunn D, Spear PG. 1989. Initial interaction of herpes simplex virus with cells is binding to heparan sulfate. J Virol 63:52–58. doi:10.1128/JVI.63.1.52-58.1989. 2535752 PMC247656

[47] Roderiquez G, Oravecz T, Yanagishita M, Bou-Habib DC, Mostowski H, Norcross MA. 1995. Mediation of human immunodeficiency virus type 1 binding by interaction of cell surface heparan sulfate proteoglycans with the V3 region of envelope gp120-gp41. J Virol 69:2233–2239. doi:10.1128/JVI.69.4.2233-2239.1995. 7884870 PMC188892

[48] Watterson D, Kobe B, Young PR. 2012. Residues in domain III of the dengue virus envelope glycoprotein involved in cell-surface glycosaminoglycan binding. J Gen Virol 93:72–82. doi:10.1099/vir.0.037317-0. 21957126

[49] Dechecchi MC, Tamanini A, Bonizzato A, Cabrini G. 2000. Heparan sulfate glycosaminoglycans are involved in adenovirus type 5 and 2-host cell interactions. Virology 268:382–390. doi:10.1006/viro.1999.0171. 10704346

[50] Giroglou T, Florin L, Schäfer F, Streeck RE, Sapp M. 2001. Human papillomavirus infection requires cell surface heparan sulfate. J Virol 75:1565–1570. doi:10.1128/JVI.75.3.1565-1570.2001. 11152531 PMC114064

[51] Hallak LK, Collins PL, Knudson W, Peeples ME. 2000. Iduronic acid-containing glycosaminoglycans on target cells are required for efficient respiratory syncytial virus infection. Virology 271:264–275. doi:10.1006/viro.2000.0293. 10860881

[52] Wang E, Brault AC, Powers AM, Kang W, Weaver SC. 2003. Glycosaminoglycan binding properties of natural Venezuelan equine encephalitis virus isolates. J Virol 77:1204–1210. doi:10.1128/jvi.77.2.1204-1210.2003. 12502837 PMC140800

[53] Tan CW, Poh CL, Sam I-C, Chan YF. 2013. Enterovirus 71 uses cell surface heparan sulfate glycosaminoglycan as an attachment receptor. J Virol 87:611–620. doi:10.1128/JVI.02226-12. 23097443 PMC3536405

[54] Lindahl U, Couchman J, Kimata K, Esko JD. 2015. Proteoglycans and sulfated glycosaminoglycans, Chapter 17. *In* Varki A, Cummings RD, Esko JD, Stanley P, Hart GW, Aebi M, Darvill AG, Kinoshita T, Packer NH, Prestegard JH, Schnaar RL, Seeberger PH (ed), Essentials of glycobiology, 3rd ed Cold Spring Harbor Laboratory Press, Cold Spring Harbor, NY. https://www.ncbi.nlm.nih.gov/books/NBK453033/.

[55] Dinglasan RR, Alaganan A, Ghosh AK, Saito A, van Kuppevelt TH, Jacobs-Lorena M. 2007. Plasmodium falciparum ookinetes require mosquito midgut chondroitin sulfate proteoglycans for cell invasion. Proc Natl Acad Sci U S A 104:15882–15887. doi:10.1073/pnas.0706340104. 17873063 PMC2000438

[56] Ciano KA, Saredy JJ, Bowers DF. 2014. Heparan sulfate proteoglycan: an arbovirus attachment factor integral to mosquito salivary gland ducts. Viruses 6:5182–5197. doi:10.3390/v6125182. 25533661 PMC4276947

[57] Gandhi NS, Mancera RL. 2008. The structure of glycosaminoglycans and their interactions with proteins. Chem Biol Drug Des 72:455–482. doi:10.1111/j.1747-0285.2008.00741.x. 19090915

[58] Kusche-Gullberg M, Kjellén L. 2003. Sulfotransferases in glycosaminoglycan biosynthesis. Curr Opin Struct Biol 13:605–611. doi:10.1016/j.sbi.2003.08.002. 14568616

[59] Rabenstein DL. 2002. Heparin and heparan sulfate: structure and function. Nat Prod Rep 19:312–331. doi:10.1039/b100916h. 12137280

[60] Sinnis P, Coppi A, Toida T, Toyoda H, Kinoshita-Toyoda A, Xie J, Kemp MM, Linhardt RJ. 2007. Mosquito heparan sulfate and its potential role in malaria infection and transmission. J Biol Chem 282:25376–25384. doi:10.1074/jbc.M704698200. 17597060 PMC2121605

[61] Kim SY, Koetzner CA, Payne AF, Nierode GJ, Yu Y, Wang R, Barr E, Dordick JS, Kramer LD, Zhang F, Linhardt RJ. 2019. Glycosaminoglycan compositional analysis of relevant tissues in Zika virus pathogenesis and in vitro evaluation of heparin as an antiviral against Zika virus infection. Biochemistry 58:1155–1166. doi:10.1021/acs.biochem.8b01267. 30698412 PMC7686953

[62] Sarrazin S, Lamanna WC, Esko JD. 2011. Heparan sulfate proteoglycans. Cold Spring Harb Perspect Biol 3:a004952. doi:10.1101/cshperspect.a004952. 21690215 PMC3119907

[63] Kato M, Wang H, Bernfield M, Gallagher JT, Turnbull JE. 1994. Cell surface syndecan-1 on distinct cell types differs in fine structure and ligand binding of its heparan sulfate chains. J Biol Chem 269:18881–18890. 8034644

[64] Ledin J, Staatz W, Li J-P, Götte M, Selleck S, Kjellén L, Spillmann D. 2004. Heparan sulfate structure in mice with genetically modified heparan sulfate production. J Biol Chem 279:42732–42741. doi:10.1074/jbc.M405382200. 15292174

[65] Hardingham T, Muir H. 1972. The specific interaction of hyaluronic acid with cartilage proteoglycans. Biochim Biophys Acta 279:401–405. doi:10.1016/0304-4165(72)90160-2. 4263642

[66] Gardner CL, Hritz J, Sun C, Vanlandingham DL, Song TY, Ghedin E, Higgs S, Klimstra WB, Ryman KD. 2014. Deliberate attenuation of chikungunya virus by adaptation to heparan sulfate-dependent infectivity: a model for rational arboviral vaccine design. PLoS Negl Trop Dis 8:e2719. doi:10.1371/journal.pntd.0002719. 24587470 PMC3930508

[67] Levitt NH, Ramsburg HH, Hasty SE, Repik PM, Cole FE, Lupton HW. 1986. Development of an attenuated strain of chikungunya virus for use in vaccine production. Vaccine 4:157–162. doi:10.1016/0264-410x(86)90003-4. 3020820

[68] Harrison VR, Eckels KH, Bartelloni PJ, Hampton C. 1971. Production and evaluation of a formalin-killed Chikungunya vaccine. J Immunol 107:643–647. 4999088

[69] Akahata W, Yang Z-Y, Andersen H, Sun S, Holdaway HA, Kong W-P, Lewis MG, Higgs S, Rossmann MG, Rao S, Nabel GJ. 2010. A virus-like particle vaccine for epidemic Chikungunya virus protects nonhuman primates against infection. Nat Med 16:334–338. doi:10.1038/nm.2105. 20111039 PMC2834826

[70] Vanlandingham DL, Hong C, Klingler K, Tsetsarkin K, McElroy KL, Powers AM, Lehane MJ, Higgs S. 2005. Differential infectivities of o’nyong-nyong and chikungunya virus isolates in Anopheles gambiae and Aedes aegypti mosquitoes. Am J Trop Med Hyg 72:616–621. doi:10.4269/ajtmh.2005.72.616. 15891138

[71] Chang L-J, Dowd KA, Mendoza FH, Saunders JG, Sitar S, Plummer SH, Yamshchikov G, Sarwar UN, Hu Z, Enama ME, Bailer RT, Koup RA, Schwartz RM, Akahata W, Nabel GJ, Mascola JR, Pierson TC, Graham BS, Ledgerwood JE, VRC 311 Study Team. 2014. Safety and tolerability of chikungunya virus-like particle vaccine in healthy adults: a phase 1 dose-escalation trial. Lancet 384:2046–2052. doi:10.1016/S0140-6736(14)61185-5. 25132507

[72] Goo L, Dowd KA, Lin T-Y, Mascola JR, Graham BS, Ledgerwood JE, Pierson TC. 2016. A virus-like particle vaccine elicits broad neutralizing antibody responses in humans to all chikungunya virus genotypes. J Infect Dis 214:1487–1491. doi:10.1093/infdis/jiw431. 27655868 PMC5091377

[73] Chen GL, Coates EE, Plummer SH, Carter CA, Berkowitz N, Conan-Cibotti M, Cox JH, Beck A, O’Callahan M, Andrews C, Gordon IJ, Larkin B, Lampley R, Kaltovich F, Gall J, Carlton K, Mendy J, Haney D, May J, Bray A, Bailer RT, Dowd KA, Brockett B, Gordon D, Koup RA, Schwartz R, Mascola JR, Graham BS, Pierson TC, Donastorg Y, Rosario N, Pape JW, Hoen B, Cabié A, Diaz C, Ledgerwood JE, VRC 704 Study Team. 2020. Effect of a chikungunya virus-like particle vaccine on safety and tolerability outcomes. A Randomized Clinical Trial. JAMA 323:1369–1377. doi:10.1001/jama.2020.2477. 32286643 PMC7156994

[74] Heinze T, Liebert T, Heublein B, Hornig S. 2006. Functional polymers based on dextran, p 199–291. *In* Klemm D (ed), Polysaccharides II. Springer, Berlin, Germany.

[75] Jones JE, Long KM, Whitmore AC, Sanders W, Thurlow LR, Brown JA, Morrison CR, Vincent H, Peck KM, Browning C, Moorman N, Lim JK, Heise MT. 2017. Disruption of the opal stop codon attenuates chikungunya virus-induced arthritis and pathology. mBio 8:e01456-17. doi:10.1128/mBio.01456-17. 29138302 PMC5686535

[76] Gorchakov R, Wang E, Leal G, Forrester NL, Plante K, Rossi SL, Partidos CD, Adams AP, Seymour RL, Weger J, Borland EM, Sherman MB, Powers AM, Osorio JE, Weaver SC. 2012. Attenuation of Chikungunya virus vaccine strain 181/clone 25 is determined by two amino acid substitutions in the E2 envelope glycoprotein. J Virol 86:6084–6096. doi:10.1128/JVI.06449-11. 22457519 PMC3372191

[77] Edelman R, Tacket CO, Wasserman SS, Bodison SA, Perry JG, Mangiafico JA. 2000. Phase II safety and immunogenicity study of live chikungunya virus vaccine TSI-GSD-218. Am J Trop Med Hyg 62:681–685. doi:10.4269/ajtmh.2000.62.681. 11304054

[78] Matusali G, Colavita F, Bordi L, Lalle E, Ippolito G, Capobianchi MR, Castilletti C. 2019. Tropism of the chikungunya virus. Viruses 11:175. doi:10.3390/v11020175. PMC6410217 30791607

[79] Chen C-L, Hasan SS, Klose T, Sun Y, Buda G, Sun C, Klimstra WB, Rossmann MG. 2020. Cryo-EM structure of eastern equine encephalitis virus in complex with heparan sulfate analogues. Proc Natl Acad Sci U S A 117:8890–8899. doi:10.1073/pnas.1910670117. 32245806 PMC7183182

[80] Xu Y, Martinez P, Séron K, Luo G, Allain F, Dubuisson J, Belouzard S. 2015. Characterization of hepatitis C virus interaction with heparan sulfate proteoglycans. J Virol 89:3846–3858. doi:10.1128/JVI.03647-14. 25609801 PMC4403428

[81] Schowalter RM, Pastrana DV, Buck CB. 2011. Glycosaminoglycans and sialylated glycans sequentially facilitate Merkel cell polyomavirus infectious entry. PLoS Pathog 7:e1002161. doi:10.1371/journal.ppat.1002161. 21829355 PMC3145800

[82] Hallak LK, Spillmann D, Collins PL, Peeples ME. 2000. Glycosaminoglycan sulfation requirements for respiratory syncytial virus infection. J Virol 74:10508–10513. doi:10.1128/jvi.74.22.10508-10513.2000. 11044095 PMC110925

[83] Kim SY, Zhao J, Liu X, Fraser K, Lin L, Zhang X, Zhang F, Dordick JS, Linhardt RJ. 2017. Interaction of Zika virus envelope protein with glycosaminoglycans. Biochemistry 56:1151–1162. doi:10.1021/acs.biochem.6b01056. 28151637 PMC7579681

[84] Lin J-Y, Shih S-R. 2014. Cell and tissue tropism of enterovirus 71 and other enteroviruses infections. J Biomed Sci 21:18. doi:10.1186/1423-0127-21-18. 24602216 PMC3995930

[85] Tseligka ED, Sobo K, Stoppini L, Cagno V, Abdul F, Piuz I, Meylan P, Huang S, Constant S, Tapparel C. 2018. A VP1 mutation acquired during an enterovirus 71 disseminated infection confers heparan sulfate binding ability and modulates ex vivo tropism. PLoS Pathog 14:e1007190. doi:10.1371/journal.ppat.1007190. 30075025 PMC6093697

[86] Esko JD, Prestegard J H, Linhardt RJ. 2015. Proteins that bind sulfated glycosaminoglycans, Chapter 38. *In* Varki A, Cummings RD, Esko JD, Stanley P, Hart GW, Aebi M, Darvill AG, Kinoshita T, Packer NH, Prestegard JH, Schnaar RL, Seeberger PH (ed), Essentials of glycobiology, 3rd ed Cold Spring Harbor Laboratory Press, Cold Spring Harbor, NY. https://www.ncbi.nlm.nih.gov/books/NBK453026/. 28876859

[87] Aquino RS, Park PW. 2016. Glycosaminoglycans and infection. Front Biosci (Landmark Ed) 21:1260–1277. doi:10.2741/4455. 27100505 PMC4975577

[88] Kamhi E, Joo EJ, Dordick JS, Linhardt RJ. 2013. Glycosaminoglycans in infectious disease. Biol Rev Camb Philos Soc 88:928–943. doi:10.1111/brv.12034. 23551941

[89] Stopschinski BE, Holmes BB, Miller GM, Manon VA, Vaquer-Alicea J, Prueitt WL, Hsieh-Wilson LC, Diamond MI. 2018. Specific glycosaminoglycan chain length and sulfation patterns are required for cell uptake of tau versus α-synuclein and β-amyloid aggregates. J Biol Chem 293:10826–10840. doi:10.1074/jbc.RA117.000378. 29752409 PMC6036193

[90] Chandra N, Liu Y, Liu J-X, Frängsmyr L, Wu N, Silva LM, Lindström M, Chai W, Pedrosa Domellöf F, Feizi T, Arnberg N. 2019. Sulfated glycosaminoglycans as viral decoy receptors for human adenovirus type 37. Viruses 11:247. doi:10.3390/v11030247. PMC6466042 30871026

[91] Bugatti A, Paiardi G, Urbinati C, Chiodelli P, Orro A, Uggeri M, Milanesi L, Caruso A, Caccuri F, D'Ursi P, Rusnati M. 2019. Heparin and heparan sulfate proteoglycans promote HIV-1 p17 matrix protein oligomerization: computational, biochemical and biological implications. Sci Rep 9:15768. doi:10.1038/s41598-019-52201-w. 31673058 PMC6823450

[92] Chen Y, Maguire T, Hileman RE, Fromm JR, Esko JD, Linhardt RJ, Marks RM. 1997. Dengue virus infectivity depends on envelope protein binding to target cell heparan sulfate. Nat Med 3:866–871. doi:10.1038/nm0897-866. 9256277

[93] Gama CI, Tully SE, Sotogaku N, Clark PM, Rawat M, Vaidehi N, Goddard WA, Nishi A, Hsieh-Wilson LC. 2006. Sulfation patterns of glycosaminoglycans encode molecular recognition and activity. Nat Chem Biol 2:467–473. doi:10.1038/nchembio810. 16878128

[94] Peerboom N, Block S, Altgärde N, Wahlsten O, Möller S, Schnabelrauch M, Trybala E, Bergström T, Bally M. 2017. Binding kinetics and lateral mobility of HSV-1 on end-grafted sulfated glycosaminoglycans. Biophys J 113:1223–1234. doi:10.1016/j.bpj.2017.06.028. 28697896 PMC5607149

[95] Tiwari V, Clement C, Xu D, Valyi-Nagy T, Yue BYJT, Liu J, Shukla D. 2006. Role for 3-O-sulfated heparan sulfate as the receptor for herpes simplex virus type 1 entry into primary human corneal fibroblasts. J Virol 80:8970–8980. doi:10.1128/JVI.00296-06. 16940509 PMC1563926

[96] Shukla D, Liu J, Blaiklock P, Shworak NW, Bai X, Esko JD, Cohen GH, Eisenberg RJ, Rosenberg RD, Spear PG. 1999. A novel role for 3-O-sulfated heparan sulfate in herpes simplex virus 1 entry. Cell 99:13–22. doi:10.1016/s0092-8674(00)80058-6. 10520990

[97] LyonS M, Deakin J, Gallagher J. 1994. Liver heparan sulfate structure. J Biol Chem 269:11208–11121. 8157650

[98] de Agostini AI, Watkins SC, Slayter HS, Youssoufian H, Rosenberg RD. 1990. Localization of anticoagulantly active heparan sulfate proteoglycans in vascular endothelium: antithrombin binding on cultured endothelial cells and perfused rat aorta. J Cell Biol 111:1293–1304. doi:10.1083/jcb.111.3.1293. 2144002 PMC2116297

[99] Herrero LJ, Foo S-S, Sheng K-C, Chen W, Forwood MR, Bucala R, Mahalingam S. 2015. Pentosan polysulfate: a novel glycosaminoglycan-like molecule for effective treatment of alphavirus-induced cartilage destruction and inflammatory disease. J Virol 89:8063–8076. doi:10.1128/JVI.00224-15. 26018160 PMC4505659

[100] Modhiran N, Gandhi NS, Wimmer N, Cheung S, Stacey K, Young PR, Ferro V, Watterson D. 2019. Dual targeting of dengue virus virions and NS1 protein with the heparan sulfate mimic PG545. Antiviral Res 168:121–127. doi:10.1016/j.antiviral.2019.05.004. 31085206

[101] Supramaniam A, Liu X, Ferro V, Herrero LJ. 2018. Prophylactic antiheparanase activity by PG545 is antiviral in vitro and protects against Ross River virus disease in mice. Antimicrob Agents Chemother 62:e01959-17. doi:10.1128/AAC.01959-17. 29437628 PMC5913976

[102] Supramaniam A, Bielefeldt-Ohmann H, Rudd PA, Webster J, Ferro V, Herrero LJ. 2019. PG545 treatment reduces RRV-induced elevations of AST, ALT with secondary lymphoid organ alterations in C57BL/6 mice. PLoS One 14:e0217998. doi:10.1371/journal.pone.0217998. 31170255 PMC6553857

[103] Hawman DW, Fox JM, Ashbrook AW, May NA, Schroeder KMS, Torres RM, Crowe JE, Dermody TS, Diamond MS, Morrison TE. 2016. Pathogenic chikungunya virus evades B cell responses to establish persistence. Cell Rep 16:1326–1338. doi:10.1016/j.celrep.2016.06.076. 27452455 PMC5003573

[104] Rohatgi A, Corbo JC, Monte K, Higgs S, Vanlandingham DL, Kardon G, Lenschow DJ. 2014. Infection of myofibers contributes to increased pathogenicity during infection with an epidemic strain of chikungunya virus. J Virol 88:2414–2425. doi:10.1128/JVI.02716-13. 24335291 PMC3958092

[105] Klimstra WB, Ryman KD, Johnston RE. 1998. Adaptation of Sindbis virus to BHK cells selects for use of heparan sulfate as an attachment receptor. J Virol 72:7357–7366. doi:10.1128/JVI.72.9.7357-7366.1998. 9696832 PMC109960

[106] Smit JM, Waarts B-L, Kimata K, Klimstra WB, Bittman R, Wilschut J. 2002. Adaptation of alphaviruses to heparan sulfate: interaction of Sindbis and Semliki forest viruses with liposomes containing lipid-conjugated heparin. J Virol 76:10128–10137. doi:10.1128/jvi.76.20.10128-10137.2002. 12239287 PMC136541

[107] Heil ML, Albee A, Strauss JH, Kuhn RJ. 2001. An amino acid substitution in the coding region of the E2 glycoprotein adapts Ross River virus to utilize heparan sulfate as an attachment moiety. J Virol 75:6303–6309. doi:10.1128/JVI.75.14.6303-6309.2001. 11413296 PMC114352

[108] Basore K, Kim AS, Nelson CA, Zhang R, Smith BK, Uranga C, Vang L, Cheng M, Gross ML, Smith J, Diamond MS, Fremont DH. 2019. Cryo-EM structure of chikungunya virus in complex with the Mxra8 receptor. Cell 177:1725–1737. doi:10.1016/j.cell.2019.04.006. 31080061 PMC7227486

[109] Liu Y, Childs RA, Palma AS, Campanero-Rhodes MA, Stoll MS, Chai W, Feizi T. 2012. Neoglycolipid-based oligosaccharide microarray system: preparation of NGLs and their noncovalent immobilization on nitrocellulose-coated glass slides for microarray analyses. Methods Mol Biol 808:117–136. doi:10.1007/978-1-61779-373-8_8. 22057521

[110] Palma AS, Liu Y, Childs RA, Herbert C, Wang D, Chai W, Feizi T. 2011. The human epithelial carcinoma antigen recognized by monoclonal antibody AE3 is expressed on a sulfoglycolipid in addition to neoplastic mucins. Biochem Biophys Res Commun 408:548–552. doi:10.1016/j.bbrc.2011.04.055. 21527252 PMC3100433

[111] Liu Y, McBride R, Stoll M, Palma AS, Silva L, Agravat S, Aoki-Kinoshita KF, Campbell MP, Costello CE, Dell A, Haslam SM, Karlsson NG, Khoo K-H, Kolarich D, Novotny MV, Packer NH, Ranzinger R, Rapp E, Rudd PM, Struwe WB, Tiemeyer M, Wells L, York WS, Zaia J, Kettner C, Paulson JC, Feizi T, Smith DF. 2017. The minimum information required for a glycomics experiment (MIRAGE) project: improving the standards for reporting glycan microarray-based data. Glycobiology 27:280–284. doi:10.1093/glycob/cww118. 27993942 PMC5444268

[112] Pal P, Dowd KA, Brien JD, Edeling MA, Gorlatov S, Johnson S, Lee I, Akahata W, Nabel GJ, Richter MKS, Smit JM, Fremont DH, Pierson TC, Heise MT, Diamond MS. 2013. Development of a highly protective combination monoclonal antibody therapy against Chikungunya virus. PLoS Pathog 9:e1003312. doi:10.1371/journal.ppat.1003312. 23637602 PMC3630103

